# Real-time reverse transcription recombinase polymerase amplification for rapid detection of murine hepatitis virus

**DOI:** 10.3389/fmicb.2022.1067694

**Published:** 2022-12-02

**Authors:** Xiao Wang, Xin Sui, Yueyu Ma, Ming Li, Xu Zhang, Dongliang Fei, Mingxiao Ma

**Affiliations:** ^1^College of Animal Husbandry and Veterinary Medicine, Jinzhou Medical University, Jinzhou, China; ^2^First Affiliated Hospital of Jinzhou Medical University, Jinzhou, China; ^3^Experimental Animal Center of Jinzhou Medical University, Jinzhou, China; ^4^The Centers for Disease Control and Prevention in Jinzhou, Jinzhou, China

**Keywords:** murine hepatitis virus, real-time, recombinase polymerase amplification, rapid detection, limit of detection

## Abstract

Murine hepatitis virus (MHV) is a highly infectious murine coronavirus that has a high potential for causing harm to host animals. This study aimed to develop a real-time reverse transcription recombinase polymerase amplification (RT-RPA) method for rapid detection of MHV in laboratory mice.

**Methods:**

Specific primers and probes for RT-RPA assay were designed targeting the conserved region in the M gene of the MHV reference strain (accession no. FJ6647223) according to the TwistDx manual instructions. The specificity, sensitivity, and reproducibility of the RT-RPA method were evaluated and compared with those of the standard RT-qPCR method. The clinical applicability of this assay was evaluated using 68 field samples.

**Results:**

Amplification using the newly developed RT-RPA assay was completed within 20 min at 37°C, while that using the RT-qPCR method required nearly 60 min. The RT-RPA method exhibited an obvious time-saving advantage. Both RT-RPA and RT-PCR methods had the same limit of detection, which was 4.45 × 10^1^ copies/μL. The specificity was indicated by a lack of cross-reaction with MHV, pneumonia virus of mice, Sendai virus, hantavirus, minute virus of mice, and reovirus type III. The MHV detection rate of RT-RPA assays was 13.63% (9/66) and RT-qPCR assays was 15.15% (10/66). Cohen’s “kappa” (κ) analysis results exhibited a very good agreement between two methods with the value of κ ≥ 0.750(since κ = 0.939) and *p* < 0.0005 (since *p* = 0.000).

**Conclusion:**

The RT-RPA assay offers an alternative tool for simple, rapid, and reliable detection of MHV in laboratory mice and has significant potential for application in laboratories.

## Introduction

Murine hepatitis virus (MHV) belongs to the coronavirus family of enveloped positive-strand RNA viruses and is one of the most prevalent viruses in laboratory mouse colonies. MHV, which contains a 31-kb single-strand positive RNA genome, is a highly mutable virus that comprises numerous antigenically distinct serotypes with differing pathological effects (Hanaki et al., 2013). MHV can be categorized into respiratory and enterotropic strains according to their tissue tropism (Kim et al., 2021). MHV infection is typically observed in newborn and weaning mice, and the major clinical manifestations include hepatitis, demyelinating encephalomyelitis, and enteritis in susceptible animals (Islam et al., 2015). According to incomplete statistics, the MHV detection rate can be as high as 30–50% in some experimental mouse colonies. MHV is a mandatory test item in the Chinese “Laboratory Animal Microbiological Standards and Monitoring” (GB14922.2–2011), which are the guidelines for specific pathogen-free (SPF)-grade laboratory rats, mice, guinea pigs, and hamsters.

Currently, indirect enzyme-linked immunosorbent assay (ELISA) of the anti-MHV antibodies is recommended as the standard detection method for MHV with certain limitations: the antibody level in mouse serum during the latent infection period may be too low to be serologically detected, and mice in immunodeficiency colonies have difficulty producing antibodies after infection. Both of these situations lead to false-negative results. Moreover, the levels of non-specific antibodies in transgenic mice may be elevated, increasing the optical density values of both specific antigens and controls, thereby leading to false-positive results in indirect ELISA. These adverse situations reduce the reliability of ELISA testing and point to the need for the development of an efficient, convenient, and stable method for the detection of MHV in laboratory mouse colonies. Currently, RT-PCR (Nakayama and Kyuwa, 2022) and real-time RT-PCR (Escutenaire et al., 2007) are commonly employed to detect MHV using nucleic acid-based molecular technology. RT-qPCR requires expensive equipment and takes more time, thus making it less suitable to use in resource-poor laboratories. A rapid, portable, and reliable on-site diagnostic assay, which is yet to be developed, would meet this need. As a novel isothermal nucleic acid amplification technology, recombinase polymerase amplification (RPA) has been demonstrated to be a rapid, specific, sensitive, and cost-effective molecular method to identify pathogens (Higgins et al., 2019). As with PCR, RPA does not require a sophisticated thermal cycler and instead can be performed at a constant temperature using a simple water bath or heating block. The entire amplification process can be completed within 20 min at 37°C–42°C (Salazar et al., 2021), which makes RPA a promising on-site detection method. Currently, RPA is successfully employed for the molecular detection of diverse pathogens, such as bacteria, fungi, parasites, and viruses, with different detection strategies (Yang et al., 2020; Arif et al., 2021; Guo et al., 2021; Kobialka et al., 2021); however, there are no reports on the application of the RPA method for MHV. Hence, in this study, fluorescent probe-based RT-RPA assay for MHV detection was developed based on the highly conserved region of the M gene. The sensitivity and specificity of the probe to detect MHV, as well as cross-detection, were determined. The assay’s clinical performance was validated using field samples, and all results were compared with real-time PCR as the reference method.

## Materials and methods

### Primer and probe design for RT-RPA

Six pairs of MHV-specific primers and two probes for RT-RPA assay were designed and synthesized according to the conserved M gene sequence in strain FJ6647223 referring to MHV (The target gene sequence comparison diagrams of M gene are in the Supplementary material). The specific primers and exo probes were generated using software Oligo7.37 and BioEdit 7.0.9. This sequence is known to exhibit good homology and conservation, and all the designed primers and probes that specifically recognized the target sequence were verified by BLASTing *via* National Center for Biotechnology Information online and then synthesized by Shanghai Sangon Co., Ltd. The details of primers and probes used in this study are shown in Table 1.

**Table 1:** The sequences of primers and probes used in the RPA assay.

No.	Name	Sequence (5′-3′)	position^a^	Amplicon size (bp)
1	M-MHV-F1	CGTTGGGCATTATACTACTCTTTATTACTATC	86–117	191
	M-MHV-R1	CAAAATACATAATCCACATTACAATGGACAC	277–307	
2	M-MHV-F2	CTACTCTTTATTACTATCATACTACAGTTCGG	100–131	235
	M-MHV-R2	GTCCTGATAAACAACCTAATGCTATTAACAAA	304–335	
3	M-MHV-F3	TACTCTTTATTACTATCATACTACAGTTCGGT	101–132	206
	M-MHV-R3	CAAAATACATAATCCACATTACAATGGACACT	276–307	
4	M-MHV-F4	CATTATACTACTCTTTATTACTATCAT	93–119	224
	M-MHV-R4	TGCTATTAACAAAATACATAATCCA	292–316	
5	M-MHV-F5	CATTATACTACTCTTTATTACTATCATACT	93–122	227
	M-MHV-R5	AATGCTATTAACAAAATACATAATCCA	292–318	
6	M-MHV-F6	CATTATACTACTCTTTATTACTATCATACT	93–122	214
	M-MHV-R6	CAAAATACATAATCCACATTACAAT	283–307	
7	Probe-MHV-1	AACACTATAGAAAATCCAAGATACACAt/i6FAMdT/a/idSp/t/iBHQ1dT/ AGCGCATACACGCAATTA/iSpC3/	243–269	
8	Probe-MHV-2	AATTGCGTGTATGCGCTAAATAATGTGTATCT-FAMdT-G-THF-A-BHQ1dT-TTTCTATAGTGTTTAC-C3 Spacer	220–251	

^a^ The position of primers and probes on the M gene of the selected sequence.

(GenBank Accession No. FJ647223).

# FAM, fluoresceinamidit; THF, tetrahydrofuran; BHQ, blackberry quencher; bp, base-pairs.

### Standard plasmid preparation for MHV quantification using RT-qRPA and RT-qPCR

The pET28b-MS2-MHV recombinant plasmid harboring the MHV gene fragment was synthesized by Jiangsu Jinweizhi Biotechnology Co., Ltd. The pET28b-MS2-MHV plasmid was recovered using the Beijing Quanshijin Biotechnology Co., Ltd. plasmid extraction kit at a final concentration of 4.45 × 10^5^ copies/μL and was used as the initial standard template for this study.

### Virus strains

MHV, pneumonia virus of mice (PVM), Sendai virus (SV), hantavirus (HV), minute virus of mice (MVM), and reovirus type III (Reo-III) cDNA were donated by Professor Bai Yingjie, Department of Laboratory Animals, Peking University. Clinical samples were provided by Jinzhou Center for Disease Control and Prevention.

### RPA assay

RPA reactions were performed in 50 μl volume using the DNA Isothermal Rapid Amplification Kit from Anpu Future Biotechnology Co., Ltd. Two microliters of forward and reverse RPA primers (10 μM final concentration), as well as 0.6 μl of exo probe (10 μM final concentration) M, were added to 12.5 μl of ddH_2_O and mixed with 29.4 μl of rehydration buffer. All reagents were prepared in a master mix, which was distributed into 0.2-mL reaction tubes containing the RPA enzyme lyophilized powder. Next, 1 μl of cDNA template was pipetted into the reaction tubes softly, and the internal and blank control templates were treated in parallel. Finally, 2.5 μl of 280 mM magnesium acetate was added to each tube, and then the tubes were covered tightly for the subsequent short spin mixing to start the reaction. The tubes were immediately placed in the QuantStudio III scanner device to continue the reaction. The fluorescence signal was collected every 30 s (end point reading) for 20 min at 37°C in real-time and increased markedly with successful amplification.

To determine the optimal reaction temperature of the RPA assay, we conducted the reaction at temperatures ranging from 35°C to 39°C for 20 min, and the amplicons were monitored using a fluorescence detector.

### Sensitivity of the RPA assay

We used ddH_2_O to dilute the pET-28b-MS2-M (MHV) standard plasmid to six concentrations at a 10-fold ratio to analyze sensitivity. The template copies after dilution ranged from 4.45 × 10^5^copies/μL to 4.45 × 10^0^ copies/μL to perform RT-RPA amplification.

At the same time RT-qPCR was being completed, one of the group standards of the Chinese Society of Laboratory Animals was referenced as a standard control in this study. The forward primer (5′ GGAACTTCTCGTTGGGCATTATACT 3′), reverse primer(5′ ACCACAAGATTATCATTTTCACAACATA 3′) and TaqMan probe (5′ FAM -ACATGCTACGGCTCGTGTAACCGAACTGT-BHQ-1 3′) were synthesized by Tsingke Biotechnology Co., Ltd. Detailed information about primers and probe used in the RT-qPCR method is shown in Table 2. The recommended 50 μl reaction volume contained 25 μl of 2× EasyTaq® PCR SuperMix (Beijing Quanshijin Biotechnology Co., Ltd), 2 μl (10 mmol/l) of each primer, 18 μl of ddH_2_O, the same standard plasmid 1 μl as RPA was used as template, and 2 μl of the probe. The reaction procedure comprised an initial denaturing step at 95°C for 30 s, followed by 40 cycles at 95°C for 5 s, and 60°C for 34 s.

**Table 2:** The sequences of primers and probe used in the RT-qPCR assay.

Name	Sequence (5′-3′)	Position^a^	Amplicon size (bp)
Forward primer	GGAACTTCTCGTTGGGCATTATACT	77–101	108
Reverse primer	ACCACAAGATTATCATTTTCACAACATA	157–184	
Probe	FAM-ACATGCTACGGCTCGTGTAACCGAACTGT-BHQ-1	123–151	

^a^The position of primers and probes on the Mouse hepatitis virus conserved sequence M protein gene.

To verify the accuracy of the two methods, each gradient was performed three times, with construction of the standard curve and comparison of the Ct values for the RT-qRPA and RT-qPCR standard curves for MHV plasmids diluted for the same serial levels.

### Specificity of the RPA assay

cDNA was used as the template for the specificity analysis of the RT-RPA assay. The assay was evaluated against a panel of pathogens considered important in mouse colonies: MHV, PVM, SV, HV, MVM, and Reo-III. These six viruses are mandatory testing pathogens for laboratory mice in the national standard. The RT-RPA method was performed as previously described in Section 2.2. Positive or negative control was included in each run.

### Validation with clinical samples

A total of 66 liver tissue samples were collected, including 46 wild mouse liver tissue samples provided by Jinzhou Center for Disease Control and Prevention and 20 SPF mouse liver tissue samples provided by Laboratory Animal Center of Jinzhou Medical University. RNA extracted from these samples was tested using the RT-RPA method. The results were compared with those obtained using RT-qPCR.

### Statistical analysis

The agreement accessing between the two methods was calculated by Cohen’s “kappa” (κ) analysis where the value of κ ≥ 0.750 and *p* < 0.0005 denotes good agreement.

## Results

### Screening of the optimal primer–probe combinations

Six pairs of RPA specific primers were screened using standard plasmid as template. The amplified products were added into 100 μl phenol solution and vortex centrifuged. After centrifugation, the centrifugation supernatant was collected and run agarose gel electrophoresis. Finally, the primers with the brightest bands and non-specific bands on the agarose-gel electrophoresis diagram were selected for the next experiment. According to the final results, the second set primer (M-MHV-F2 and M-MHV-R2) was selected and used in the experiment. In the same way, the optimal probe (Probe-MHV-2) was achieved and used in the experiment according to the optimal primer.

### Optimization of amplification temperature

**Figure 1:**
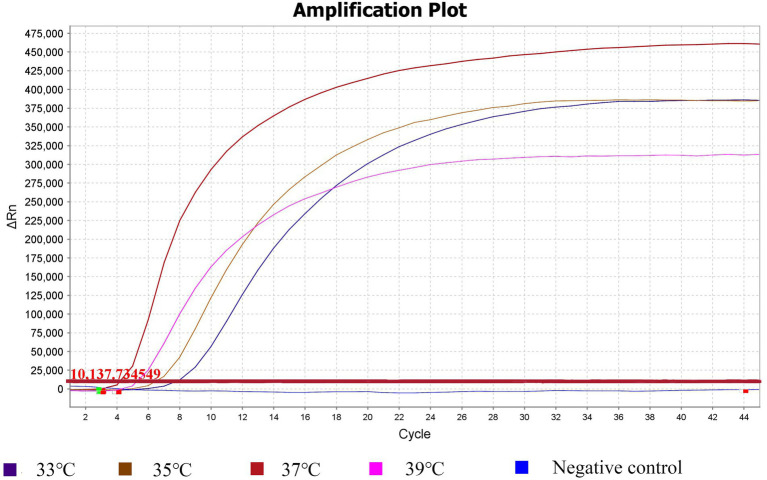
Temperature optimization for the RPA assay. RPA assay was conducted at a broad range of amplification temperatures (33°–39°C) to analyze the optimal reaction temperature for RPA reactions.

To determine the optimal reaction temperature, the RPA assay was conducted at temperatures ranging from 33°C to 39°C. Figure 1 shows that the Ct values at 33°C–39°C were 4.83, 4.24, 2.95, and 3.87. The RPA reaction was more efficient at 37°C, and the Rn value was also higher at that temperature. The optimal temperature for the RPA reaction was determined to be 37°C.

### Analytical specificity and sensitivity

**Figure 2:**
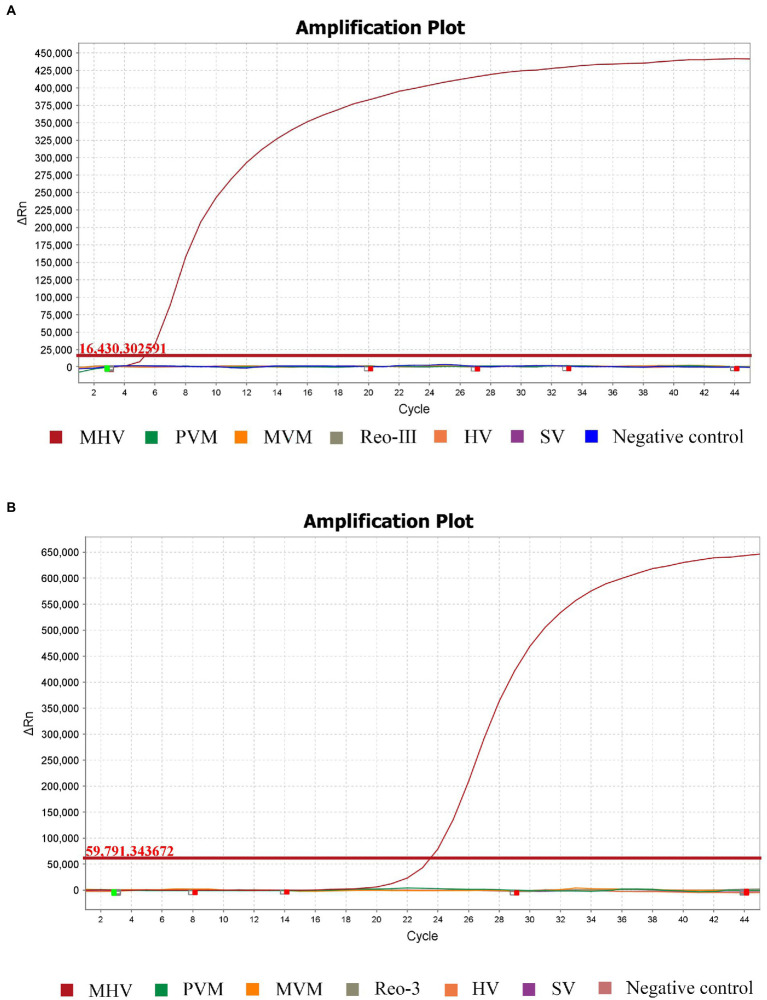
Specificity of the RPA method compared with the standard RT-qPCR. Analytical specificity of the RPA assay **(A)**. Specific fluorescence signal was observed from MHV, whereas no signals were obtained from other pathogens or the control. The same results occurred in RT-qPCR **(B)**.

Using MHV, PVM, SV, HV, MVM, and Reo-III cDNA as templates, the RT-RPA amplification reaction was performed. The results showed that only MHV was detected using the RT-RPA method and not the other viruses (Figure 2A). No cross detections were observed. The finding demonstrates the specificity of RT-RPA assay for the detection of MHV. Moreover, RT-RPA had the same MHV detection rate as RT-qPCR, as shown in Figure 2B.

**Figure 3:**
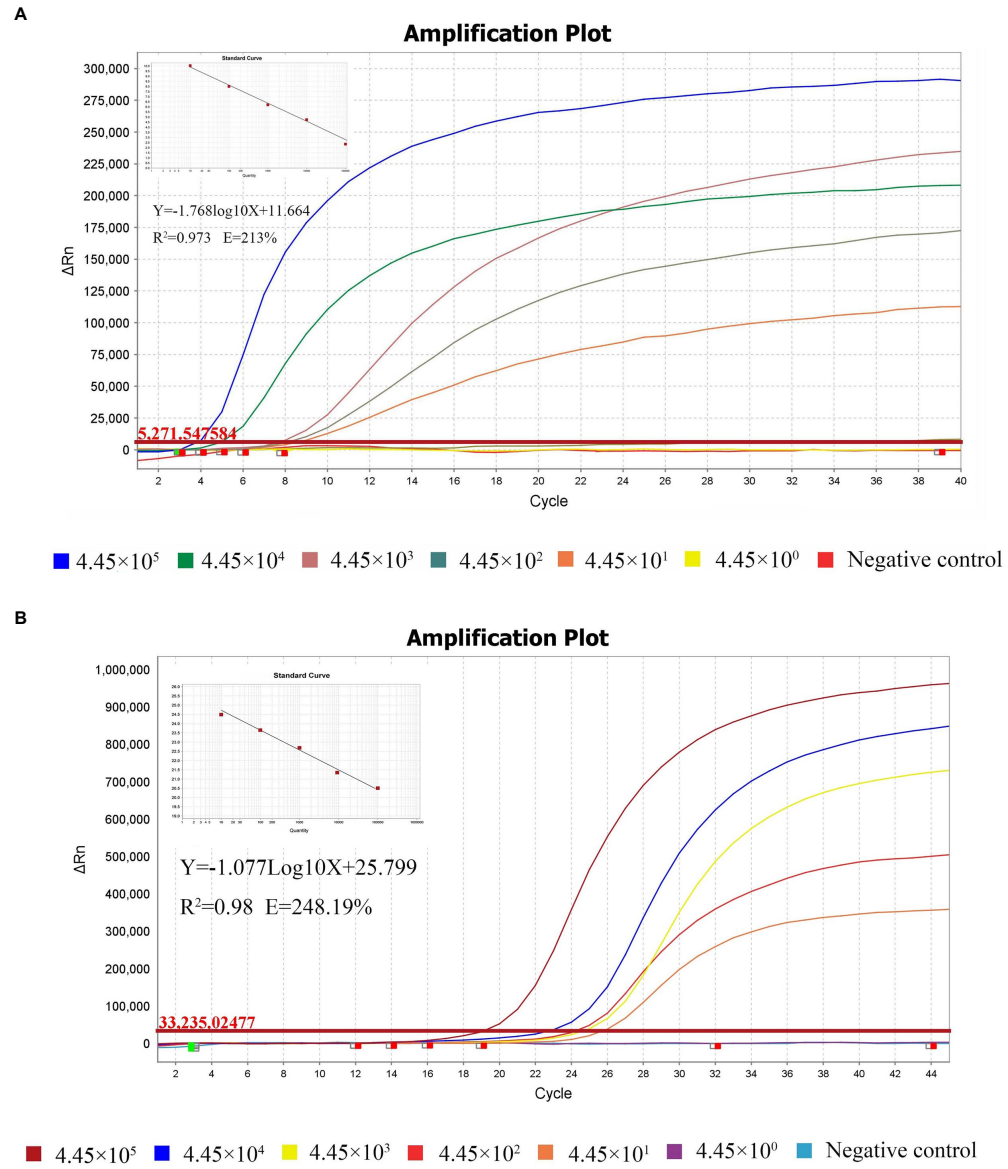
Sensitivity of the RT-qRPA method compared with the reference RT-qPCR. Analytical sensitivity of the RPA assay. Sensitivity evaluation was conducted with 10-fold dilutions of templates ranging from 4.45 × 10^5^ copies/μL to 4.45 × 10^0^ copies/μL. The LOD of both assays was 4.45 × 101 copies/μL **(A, B)**.

The sensitivity comparison of the RT-qRPA and RT-qPCR results are shown in Figures 3A,B. To evaluate sensitivity, the standard plasmid pET-28b-MS2-M (MHV) was diluted in 10-fold serial dilutions to achieve concentrations ranging from 4.45 × 10^5^ copies/μL to 4.45 × 10^0^ copies/μL in 6 gradients, for the reaction to be performed. Figures 3A,B demonstrate that both limits of detection (LOD) were 4.45 × 10^1^ copies/μL. For comparison of Ct values, MHV plasmids were diluted to the same serial level in both RT-qRPA and RT-qPCR standard curves (Table 3).

**Table 3:** Comparison of Ct values in MHV plasmids diluted for the same serial level in RT-qRPA and RT-qPCR standard curves.

^a^Initial concentration of quantified MHV plasmids	RT-RPA			Ct mean ± sd	RT-qPCR			Ct mean ± sd
	Ct1	Ct2	Ct3		Ct1	Ct2	Ct3	
4.45 × 10^5^	2.37	2.65	2.91	2.64 ± 0.27	14.52	16.12	15.72	15.45 ± 0.83
4.45 × 10^4^	3.43	3.12	4.21	3.59 ± 0.56	16.73	18.12	17.57	17.47 ± 0.70
4.45 × 10^3^	6.26	6.31	5.97	6.18 ± 0.18	20.02	19.18	19.89	19.70 ± 0.45
4.45 × 10^2^	7.97	7.92	7.82	7.90 ± 0.08	21.33	21.74	21.72	21.60 ± 0.23
4.45 × 10^1^	8.22	8.17	8.36	8.25 ± 0.10	23.77	22.82	25.71	24.10 ± 1.47
4.45 × 10^0^	—	—	—		—	—	—	
Blank	—	—	—		—	—	—	

– represents the unidentified value or invalid calculation result.

^a^Initial MHV plasmid genome copy (GC) from quantified pET28b-MS2-MHV plasmid template after a series of 10-fold dilutions in triplicate.

### Evaluation of RT-RPA with clinical samples

A total of 66 samples were tested for MHV using the RT-RPA assay and compared with RT-qPCR. Of these samples, 9 MHV-positive samples and 57 MHV-negative samples were confirmed by RT-RPA. All samples were detected by RT-qPCR, the group standards of Chinese Laboratory Animal Society. The RT-qPCR result showed that 10 samples were MHV-positive and 56 samples were MHV-negative. It should be noted that all 20 samples provided by Laboratory Animal Center of Jinzhou Medical University were negative by RT-RPA and T-qPCR test, repectively.

The amplified products were purified and cloned into pEasy-T1 vector for sequencing. The result demonstrated that the sample were positive for MHV. As shown in Table 4, Cohen’s “kappa” (κ) analysis results exhibited a very good agreement between two methods with the value of κ ≥ 0.750(since κ = 0.939) and *p* < 0.0005 (since *p* = 0.000).

**Table 4:** Detection of MHV in samples using the RT-RPA and real-time RT-PCR methods^a^.

		RT-qPCR			CR
		Positive	Negative	Total	
RT-RPA	Positive	9	0	9	98.5%
	Negative	1	56	57	
	Total	10	56	66	

^a^κ = 93.9%, *p* < 0.0005, It was statistically significant, output by Kappa Test in SPSS software.

## Discussion

MHV, first discovered in 1947, is an enveloped virus with a positive-sense, single-stranded RNA genome (Perlman, 1998; Weiss and Navas-Martin, 2005). Depending on the specific strain, MHV may infect the liver and/or the brain of mice, thus permitting its use in the study of hepatitis as well as neurological diseases (MacPhee et al., 1985). Polytropic strains, such as MHV-JHM and MHV-A59, first infect the respiratory system before spreading to other organs, whereas enterotropic strains, such as MHV-DVIM, replicate in the intestinal tract (Homberger, 1997). In SPF grade mice, MHV can produce a high level of immune response in the infected mice body and severely interfere with animal experiments. At the same time, the virus is infrequently expressed in SPF grade mice and is difficult to eradicate from infected mice. MHV is one of the pathogens that must be absent in laboratory mice of SPF grade or higher. The national standard recommends indirect ELISA for detecting serum antibodies, while the group standards of the Chinese Society of Laboratory Animals recommends RT-qPCR for detecting antigen. However, ELISA antibody detection is not always feasible, and RT-qPCR has limitations when applied in resource-poor conditions because they require professional equipment and technicians and are time-consuming. And in the direction of developing a field-portable detection tool, the RT-qPCR method seems going to be very limited. Although those shortcomings, it has been proved that RT-qPCR recommended by the Chinese Society of Laboratory Animals has high sensitivity and specificity, which can meet the quality detection requirements of experimental animals (T/CALAS 25-, 2017). Therefore, the RT-qPCR recommended by the Chinese Society of Laboratory Animals was used as the reference standard to establish the RT-RPA method in this study. The prominent advantage of the RPA assay is its rapidity and simplicity, which contributes to the faster reporting of the results. This study establishes that RT-qRPA is an important supplemental method for MHV detection. We used theTwistDx kit, which can be used to complete the reverse transcriptase and the isothermal amplification with an RNA template in one step. Hence, the established RT-qRPA assay may serve as an effective MHV detection method for monitoring the quality of laboratory animals.

The design of primers and probes is the key to the detection of RT-RPA, which may be directly related to the specificity and sensitivity of RT-RPA. RPA is tolerant to 5–9 mismatches in the primer and probe, showing no influence on the performance of the assay (Daher et al., 2015; Wang et al., 2017). Six pairs of MHV-specific primers and two probes for RT-RPA assay targeting the M gene (FJ6647223) in this study were designed by comparing the genomic sequences of different MHV strains. The best set was achieved by multiple screening and used in the experiment. There were only 1–3 mismatches in the primers and probe with other MHV strains. The specificity evaluation results of the RT-qRPA method confirmed that amplification occurred exclusively in the MHV positive control group and not in the other mouse virus templates (Figure 2A). This finding is in agreement with that of the RT-qPCR recommended as the group standards by the Chinese Society of Laboratory Animals (Figure 2B). However, longer primers of at least 30 bases and a marked exo probe of at least 45 bases in the RT-qRPA method suggest that it is more specific than in the RT-qPCR. In the sensitivity test, the LOD of the RT-RPA method reached 44.5 copies/reaction by diluting the pET-28b-MS2-M (MHV) plasmid template (Figure 3A), which agreed with the LOD of the group standards of the Chinese Society of Laboratory Animals RT-qPCR (Figure 3B). The RT-RPA assay for MHV exhibits several comparative advantages over the reference RT-qPCR. First, the RT-RPA assay is much faster than RT-qPCR for the detection of MHV (Table 3). The entire amplification process can be completed within 20 min, whereas RT-qPCR usually takes approximately 60 min. Second, the RT-RPA assay can be performed at a constant temperature between 37°C and 42°C, which is easily achieved using a simple water bath or heating block or even with body temperature. Lastly, the RT-RPA assay has the potential to be developed as an RPA-lateral flow dipstick (RPA-LFD) or a microfluidic chip format for multiplex detection.

We validated the clinical applicability of RT-RPA by evaluating its diagnostic performance for specimens. In this study, the diagnostic performance of the newly developed RT-RPA assay was comparable to that of RT-qPCR. Cohen’s “kappa” (κ) analysis results exhibited a very good agreement between two methods with the value of κ ≥ 0.750(since κ = 0.939) and *p* < 0.0005 (since *p* = 0.000). Hence, the results indicate that the established RT-RPA method is effective for detecting MHV nucleic acids and is comparable in efficacy to the reference RT-PCR method. The established RT-RPA method has short detection time and good detection effect, which provides the possibility for clinical rapid detection of MHV infection.

## Conclusion

We developed a new RT-RPA method that is highly sensitive and specific for MHV at 37°C. Amplification using RT-RPA required approximately 20 min, and its detection limit was 44.5 copies/μL. The field application performance of the method was comparable to that of the reference RT-qPCR. These practical data provide useful information for the development of a point-of-care testing method to detect MHV in laboratory animals.

## Data availability statement

The original contributions presented in the study are included in the article/Supplementary material, further inquiries can be directed to the corresponding authors.

## Ethics statement

The use of the experimental animals involved in the manuscript is in compliance with the relevant provisions of the Animal Welfare and Ethics of Experimental Animals of the Experimental Animal Center of Jinzhou Medical University, China.

## Author contributions

MM and DF: designed this study. XW, YM, XS, and ML: performed the experiments. XZ and XW: analyzed the data. All authors approved the final version of the manuscript.

## Funding

This work was supported by grants from the National Natural Science Foundation of China (32172789 and 31972626), Liaoning Provincial Natural Science Foundation of China (2020-MS-300).

## Conflict of interest

The authors declare that the research was conducted in the absence of any commercial or financial relationships that could be construed as a potential conflict of interest.

## Publisher’s note

All claims expressed in this article are solely those of the authors and do not necessarily represent those of their affiliated organizations, or those of the publisher, the editors and the reviewers. Any product that may be evaluated in this article, or claim that may be made by its manufacturer, is not guaranteed or endorsed by the publisher.
