# Supplementary material for: Real-time reverse transcription recombinase polymerase amplification for rapid detection of murine hepatitis virus
Source: Front Microbiol. 2022 Dec 2;13:1067694. doi: 10.3389/fmicb.2022.1067694 (PMC9755729; doi:10.3389/fmicb.2022.1067694)
Supplement: Supplementary file 1 [file Table_1.DOCX]

**SUPPLEMENTARY MATERIAL**

**The target gene sequence of M gene:**

A


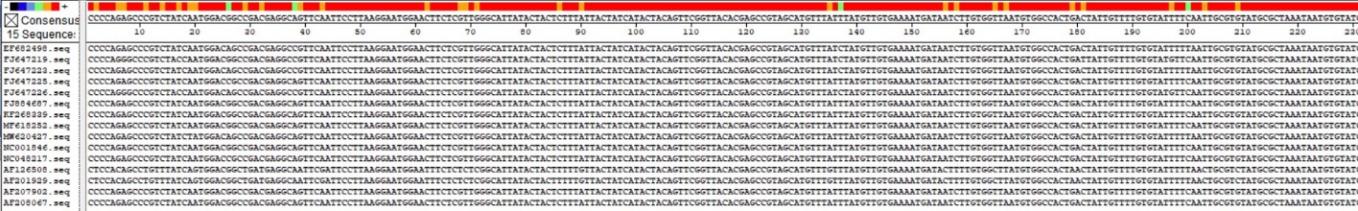


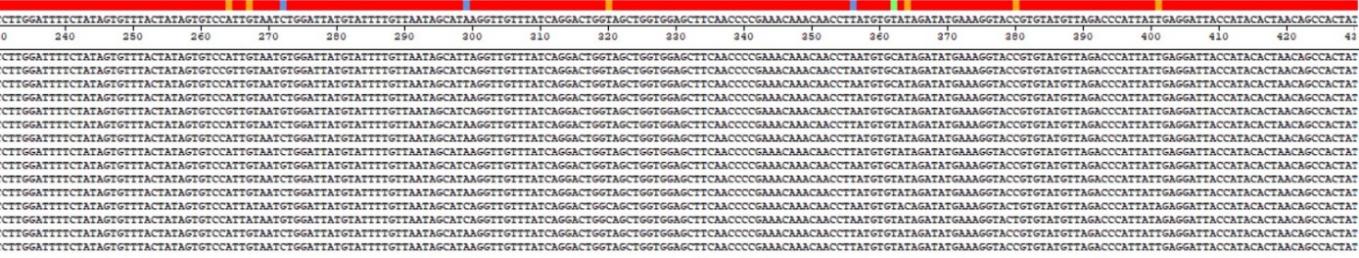


B


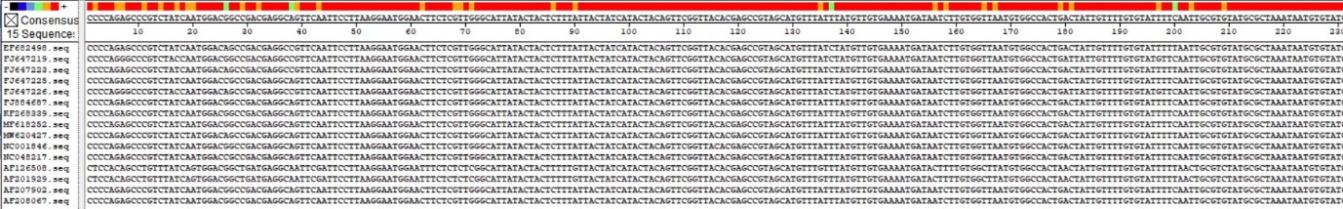


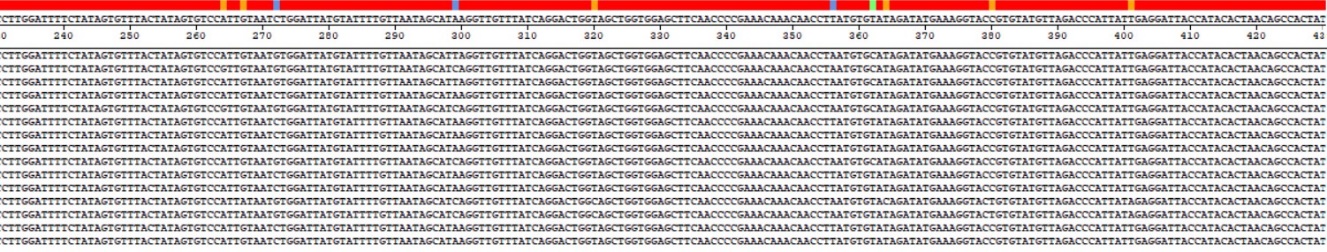


We selected several representative MHV virus strains to compare the target genes of the M gene. Nucleotide residues that match the majority are indicated by dots. The three boxes represent the forward primer, probe, and reverse primer from front to back. (A) Primers and probes used for the RT-qRPA assay. (B) Primers and probes used for the RT-qPCR assay.

GenBank accession numbers: Murine hepatitis virus-1(EF682498.1), Murine coronavirus RJHM/A(FJ647219.1), Murine coronavirus MHV-1(FJ647223.1),Murine coronavirus A59(FJ647225.1), Murine coronavirus MHV-JHM.IA(FJ647226.1), Murine hepatitis virus strain A59(FJ884687.1), Murine coronavirus A59(KF268339.1), Murine hepatitis virus A59(MF618252.1), Murine coronavirus MHV-3(MW620427.1),Murine hepatitis virus A59(NC001846.1), Murine hepatitis virus A59(NC048217.1), Murine hepatitis virus strain 2(AF126508.1),Murine hepatitis virus strain 2(AF201929.1),Murine hepatitis virus strain ML-11(AF207902.1), Murine hepatitis virus ML-10(AF208067.1).
